# Viroscope™: a universal solution for plant virus and viroid diagnostics using HTS and cloud-based analysis

**DOI:** 10.3389/fmicb.2025.1609663

**Published:** 2025-07-03

**Authors:** Verónica Morgante, Juan Cristóbal Jiménez, Claudio Ponce, Cristóbal Urrutia, Fernanda Vera, Camila Farías, Rocío Camps de la Maza, Valentina Caro, Marco Muñoz, Bernardo Pollak

**Affiliations:** ^1^Multiplex SpA, Santiago, Chile; ^2^Servicio Agrícola y Ganadero (SAG), Santiago, Chile

**Keywords:** plant health, high-throughput sequencing (HTS), border security, phytosanitary quarantine, RT-qPCR, genome assembly coverage, replicase identification, Viroscope

## Abstract

Border biosecurity and food supply face significant global challenges due to the increasing threat of plant viruses, exacerbated by international plant trade. While high-throughput sequencing (HTS) -based virus diagnosis offers promising applications, challenges in data analysis and implementation have limited widespread adoption. Viroscope™ addresses these limitations through an advanced cloud service that leverages HTS for high-certainty virus and viroid identification. A field study was conducted on plants in post-entry quarantines using the Viroscope™ algorithm to evaluate its performance for phytosanitary diagnostics of virus and viroids. Tissue samples provided by the Chilean phytosanitary agency were processed and deep-sequenced (*n* = 144) using the Illumina® platform, with parallel analysis using conventional and RT-qPCR methods. The results demonstrated the enhanced detection capabilities and biological insights by Viroscope**™** algorithm, even in cases of low viral abundance. From the tested plants in post-entry quarantine programs, 28.5% contained regulated and/or emergent viruses and viroids. No viral pathogens from the quarantine list were detected, in agreement with RT-qPCR results. Notably, 25% of plants harbored emergent viruses with functional evidence, highlighting potential risks undetected by traditional procedures. Comparative analysis with RT-qPCR confirmed that Viroscope™ results exhibited a high degree of correlation with current methods and furthermore, Viroscope™ was able to detect viruses in samples which yielded negative RT-qPCR results. Universally applicable across plant tissue, Viroscope™ detects all known viruses and viroids in public databases while employing innovative metrics for functional assessment. The cloud-based platform facilitates global adoption of HTS technology by phytosanitary agencies through user-friendly reports that enable rapid and informed decision-making.

## Introduction

Plant health is fundamental to global food security and economic stability. The exponential increase in international plant trade has intensified phytosanitary challenges, with plant diseases costing the global economy approximately $220 billion USD annually (Hantula et al., 2014; Savary et al., 2019; FAO, 2021; Jones, 2021). Coordinated through the International Plant Protection Convention (IPPC), current regulations aim to facilitate trade while minimizing pathogen dissemination through evidence-based pest risk analysis. However, increasing trade volumes coupled with climate change events require enhanced border protection strategies for effective biosecurity (Porkka et al., 2013; Ristaino et al., 2021).

Historical epidemiological events demonstrate the devastating socioeconomic consequences of inadequate plant health management and insufficient phytosanitary controls (Powderly, 2019; Folimonova and Sun, 2022; Raparelli et al., 2024). Among agricultural threats, plant viruses and viroids are particularly destructive, accounting for approximately 50% of plant diseases worldwide and over $30 billion USD in annual economic losses (Rodríguez-Verástegui et al., 2022; Jones, 2021). This impact is further exacerbated by underdiagnosis and climate change effects. Notable examples include *Citrus tristeza virus* (CTV), which destroyed over 100 million citrus plants globally (Moreno et al., 2008; Folimonova and Sun, 2022), and *Tomato brown rugose fruit virus* (ToBRFV), causing yield losses up to 70% (Oladokun et al., 2019). Current import practices rely on conventional detection methods with significant limitations: biological indexing lacks sensitivity for asymptomatic cases, ELISA shows false negative rates of 20–30%, and PCR-based methods only detect previously characterized viruses (Mirmajlessi et al., 2015; Pallás et al., 2018).

Once infected, plants harbor viral pathogens throughout their life cycle, often without evident symptoms. Current import practices rely on strict phytosanitary customs in which plants are monitored and tested using conventional detection methods (Mehetre et al., 2021). In this regard, conventional diagnostic approaches face limitations and design bias. The visual inspection and traditional bioassays are highly time-consuming and uncertain for asymptomatic cases. While ELISA testing offers cost-effectiveness at $5–10 per sample, results show false negative rates of 20–30% in early infection stages (Boonham et al., 2014). Even the gold standard PCR-based methods, despite higher sensitivity, only detect previously characterized viruses and are limited in multiplexing capacity (Mirmajlessi et al., 2015; Pallás et al., 2018). Under quarantine schemes, these limitations create substantial risk, as undetected viruses can establish new diseases in virus-free regions.

Post-entry quarantine programs (PEQ) face mounting challenges balancing phytosanitary security with trade efficiency (Martin et al., 2016; Jones, 2021; Whattam et al., 2021). These challenges are exacerbated by lack of harmonized technical capacities among governmental entities, increasing volume of international plant trade, and disruptions to global supply chains often imposing economic impacts of up to USD 5,000 per consignment. In Chile, the Agricultural and Livestock Service (SAG) implements quarantine periods extending up to 2 years for priority pests (Supplementary Table S1), following international guidelines.

High-throughput sequencing (HTS) technologies introduce promising opportunities for phytosanitary diagnostics by providing detection of multiple pathogens (Massart et al., 2014; Massart et al., 2017; Rott et al., 2017; Adams et al., 2018; Rivarez et al., 2021; Haegeman et al., 2023). HTS is particularly suitable for plant virus detection since most viral pathogens have RNA genomes and DNA viruses encode RNA intermediates for their replication. Due to its unbiased and hypothesis-free approach, total-RNA HTS implementation is both analytically robust and cost-effective for plant diagnostic applications (Maree et al., 2018; FAO, 2019; Gaafar et al., 2021; Rong et al., 2023). Despite clear advantages and detailed recommendations to facilitate HTS adoption in plant pest routine diagnostics (Lebas et al., 2022; Massart et al., 2022), implementation in regulatory frameworks still faces computational and bioinformatics issues including the need for consistent interpretation schemes, clear detection thresholds, and consensus on validation standards (Rott et al., 2017; Valenzuela et al., 2022; Hu et al., 2023; Maina et al., 2024; Ning et al., 2024). These challenges encompass critical steps such as removing low-quality sequences, eliminating plant sequences, correctly assigning remaining sequences to viral genomes, *de novo* assembly, and read mapping strategies.

**Figure 1:**
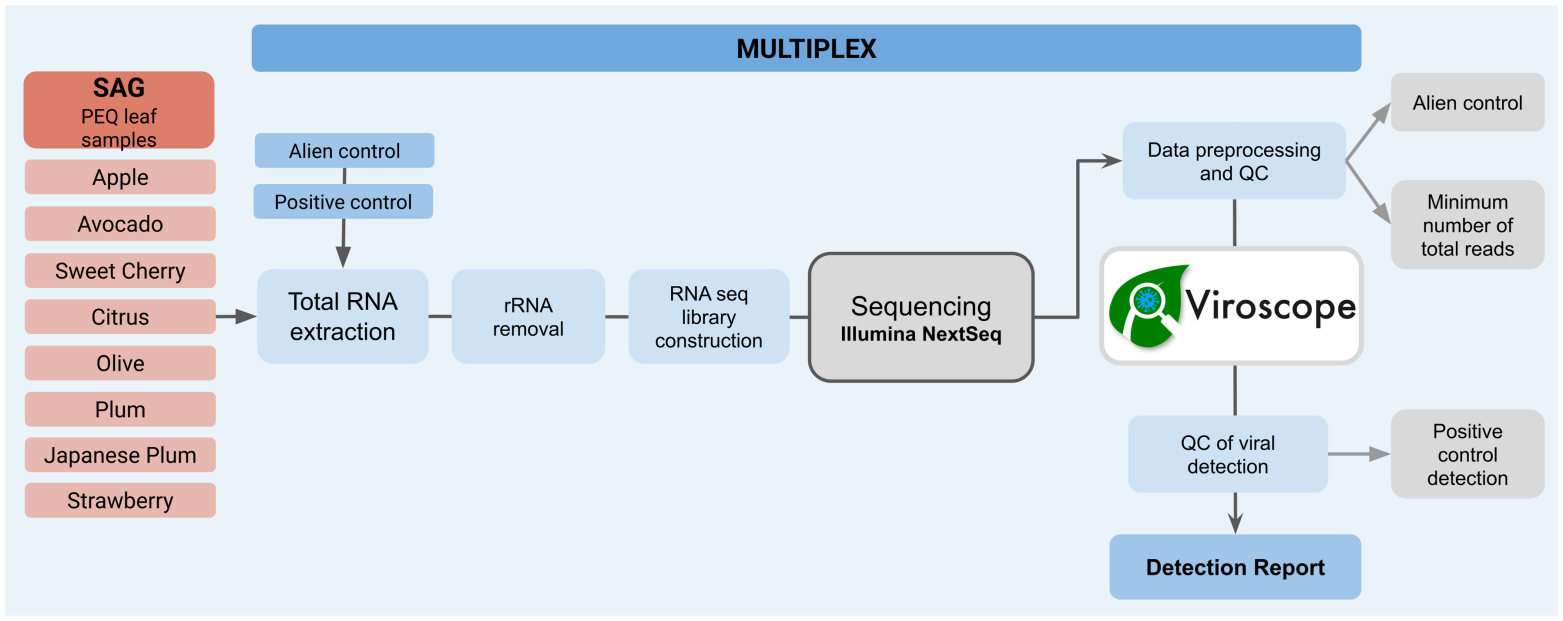
Methodology of pilot study for detection of virus and viroids from post entry quarantine (PEQ) plants using RNA seq and Viroscope™ platform. Leaf samples were processed together with alien and positive control samples and subjected to RNA extraction and RNAseq library construction. After sequencing, samples below the threshold of minimum total reads were discarded from analysis. Additionally, reads of alien control throughout the samples were considered to establish minimum read thresholds for viral analysis. Reads passing QC steps were then analyzed with the Viroscope™ algorithm, generating individual reports for each sample, containing data for genome completeness and functional evidence detection for detected viral taxa. The presence of five viruses in the previously characterized positive control sample was also verified in each sequencing run.

**Figure 2:**
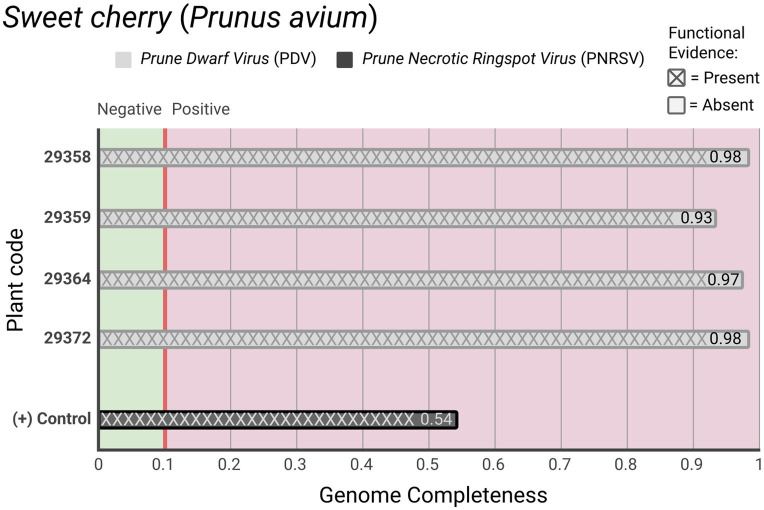
Detection of regulated viruses from PEQ samples. Genome completeness of regulated viruses detected in four plant samples of sweet cherry and the positive control. Hatched bars show cases in which viral functional evidence is also detected.

Viroscope™ directly addresses these limitations through a unique approach combining HTS data analysis with biological functionality assessment. This algorithm evaluates both genomic coverage metrics and viral replication signatures, with previous validation demonstrating superior fitness parameters (>99.9% specificity, sensitivity, and false rate discovery) for Viroscope™ compared to conventional methods (Valenzuela et al., 2022). As reported, the identification of biological signatures of the viral biology (functional annotation) have greatly increased the certainty of viral detection in real-world scenarios, particularly in cases of low abundance and low genome coverage. Here, we present a field validation in a regulatory context (PEQ) through collaboration with the Chilean phytosanitary authority (SAG), focusing on essential criteria for regulatory adoption: high certainty diagnostic, reproducibility, cost-effectiveness, instrumental implementation, and potential reduction of quarantine periods. This validation addresses bioinformatics analysis and interpretation issues by reducing labor-intensive analysis, eliminating inconsistent outcomes, and ultimately avoiding subjective decision-making in phytosanitary contexts (see Figures 1, 2).

## Materials and methods

### Plant material, sample collection, and study design

PEQ surveillance material (*n* = 219) was selected based on both regulatory priorities and economic significance within the Chilean agricultural sector. The sampling strategy included eight economically significant fruit tree species with varying sample sizes reflecting their relative importance in export markets and vulnerability to viral pathogens: *Prunus avium* (sweet cherry, *n* = 16), *Prunus domestica* (plum, *n* = 18), *Prunus salicina* (Japanese plum, *n* = 51), *Citrus* spp. (*n* = 3), *Fragaria × ananassa* (strawberry, *n* = 53), *Malus domestica* (apple, *n* = 25), *Olea europaea* (olive, *n* = 21), and *Persea americana* (avocado, *n* = 32). This distribution was designed to provide comprehensive coverage of the major fruit crops under post-entry quarantine monitoring in Chile while enabling statistical robustness for viral surveillance across taxonomically diverse hosts. All samples were collected between January–April 2023. Upon arrival, visual inspection and quality control were performed with 30 samples being excluded due to tissue necrosis or oxidative degradation. SAG subjected all plant quarantine material used in this study to strict biosecurity protocols. Following the sampling and analysis performed by Viroscope™, the plant materials were incinerated and disposed of according to established phytosanitary procedures for quarantine specimens, ensuring complete elimination of potential pathogen sources. This rigorous containment and disposal protocol safeguarded the local phytosanitary security throughout the validation process, in compliance with national and international quarantine regulations.

### Sample processing and quality assessment

Foliar tissue was homogenized using sterile pre-cooled (−20°C) mortar and pestle and total RNA was extracted using the Spectrum™ Plant Total RNA Kit (Sigma-Aldrich) following manufacturer’s protocols. Residual genomic DNA was removed using DNase I (RNAse free) (New England Biolabs). RNA quantity and quality were assessed via fluorometric quantification (Promega Quantus), spectrophotometric analysis (BioTek Synergy H1), and integrity evaluation through denaturing agarose gel electrophoresis. Host ribosomal RNA was selectively depleted using the QIAseq FastSelect rRNA Plant Kit (QIAGEN). Samples with insufficient RNA yield and quality (≥70 ng/μL, RIN value ≥6.5) were excluded, resulting in 179 specimens proceeding to sequencing library preparation.

### RNA-seq library preparation and sequencing

RNA-seq libraries were constructed using the NEBNext Ultra II RNA Library Prep Kit for Illumina (New England Biolabs). The workflow included reverse transcription, double-stranded cDNA synthesis, and enzymatic adapter ligation with sample-specific molecular barcodes incorporated via PCR amplification. DNA cleanup steps were performed using an Opentrons OT-2 liquid handling platform equipped with magnetic modules (Opentrons Labworks, Long Island City, NY, USA). Libraries were quantified using the QuantiFluor DNA System (Promega). Pooled libraries (*n* = 35 per run) were sequenced on an Illumina NextSeq 1,000 platform using P1 chemistry with paired-end 100 -cycle parameters (2 × 50 cycles). Average sequencing depth requirement was set at 14 million reads per sample. To determine the appropriate sequencing depth for pathogen detection, a control sample, SS-S2 (Valenzuela et al., 2022), was sequenced at a depth of x15M. After filtering for high-quality reads, approximately 14 million reads remained. A random subsampling of 5, 10, 30, 50, and 80% of the reads was performed, resulting in datasets of 700 K, 1.4 M, 4.2 M, 7 M, and 11.2 M reads, respectively. Subsampling was conducted using seqkit’s (Shen et al., 2016) sample function, ensuring paired-read correspondence through the seed parameter. These subsampled datasets were analyzed using Viroscope™, from which the viral genome assembly coverage (VGAC) and the number of reads assigned to each virus were determined (Supplementary Figure S1) (Soltani et al., 2021). The results indicate that when applying a 10% of the virus genome completeness criteria (VGAC threshold of 0.1), all viruses present are detected if at least 7 million reads are available. In the case of viroids, the sequencing depth defined based on viral detection was more than sufficient to achieve near-complete genome coverage. Considering that viroids range in length from 246 to 401 nucleotides (Shrestha and Bujarski, 2020), even low read counts were able to capture more than 40% of the viroid genome completeness (VGAC threshold of 0.4), as shown in Supplementary Figure S2.

### Quality control framework

For systematic validation purposes, a comprehensive analytical framework was implemented to ensure diagnostic reliability and reproducibility, adhering to established HTS diagnostic standards (Lebas et al., 2022; Massart et al., 2022; Rong et al., 2023). The validation consisted of a hierarchical control system with three independent control tiers. First, a well-characterized positive control specimen sweet cherry tree sample SS-S2 harboring multiple viral infections including *Prunus necrotic ringspot virus* (PNRSV) was included as described by Valenzuela et al. (2022). Second, a pathogen-free negative control was processed in parallel to monitor potential false positives. Third, an exogenous synthetic viroid RNA control was spiked into *P. americana* leaf tissue matrix to assess cross-contamination and establish minimum threshold parameters for reliable read assignment. Additional validation was achieved through internal positive controls and rigorous sequencing depth requirements exceeding 10 million reads per sample, ensuring adequate coverage for reliable viral detection and genome assembly.

### Bioinformatic analysis pipeline, metrics and threshold determination

A cloud-based HTS data analysis platform for virus diagnosis, Viroscope.io, was used to enhance detection accuracy through biologically-informed viral genome assembly coverage (Valenzuela et al., 2022). Briefly, the samples yielding fewer than 14 million reads (*n* = 34) were excluded from further analysis, leaving 144 samples for comprehensive assessment. The analytical pipeline began with stringent pre-processing and quality filtration, including removal of low-quality sequences ^1^, demultiplexing, adapter sequence removal and trimming via Trimmomatic (Bolger et al., 2014), host sequence depletion, and synthetic control analysis to assess cross-contamination. Following quality control, viral read assignment and assembly proceeded through a sequential workflow utilizing the Centrifuge, Kraken2, and Minimap2 platforms for viral read classification, followed by *de novo* assembly of virus-assigned reads to generate contiguous sequences, and reference-based mapping of assembled contigs for completeness assessment calculation. Based on these sequential workflows, genome coverage metrics were calculated using standardized thresholds (≥10% for viruses; ≥40% for viroids) to establish positive detection parameters. These specific thresholds were derived from previous validation studies (Valenzuela et al., 2022) which demonstrated that these values represent optimal diagnostic cutoffs balancing sensitivity and specificity, with strong correlation to the presence of viral functional elements. In viroids, the higher threshold requirement (≥40%) reflects their smaller genome size and distinctive circular RNA structure, which necessitates more stringent coverage parameters for reliable detection. The assembly coverage, viral abundance, replicase identification and general statistics of the sequencing runs assisted with the interpretation of results as reported (Rott et al., 2017; Gutiérrez et al., 2021; Valenzuela et al., 2022). The final confirmation was achieved through functional evidence assessment by identifying viral replicase sequences using the RVDB-prot database on the assembled contigs. This functional signature provided additional high-certainty confirmation of viral presence and potential infectivity while maintaining analytical stringency through multiple independent validation steps. To facilitate molecular diagnosis in post-entry quarantine scenarios under regulatory frameworks, viral read assignment was conducted against two sequential reference databases: an initial screen against quarantine and quality-control viral sequences, followed by comprehensive analysis using an expanded database encompassing all currently characterized viral genomes. Results were reported as “positive” in samples meeting the established coverage thresholds, with additional classification as “Functional Evidence = Present” for viral genomes with assembled transcripts for viral replicases.

### Comparative RT-qPCR analysis

RT-qPCR analysis was conducted on a subset of 33 samples to assess diagnostic concordance and detection sensitivity between Viroscope™ and published methods, utilizing the same RNA used for RNA-Seq library construction. Four representative viruses were targeted: *Apple stem pitting virus* (ASPV), *Cherry virus A* (CVA), *Plum bark necrosis stem pitting-associated virus* (PBNSPaV), and *Olive leaf yellowing associated virus 1* (OLYaV), using previously validated primer sets (Nabi et al., 2018; Valenzuela et al., 2022; Sabanadzovic et al., 1999). Selection criteria included taxonomic diversity across host species, availability of control specimens, and existence of validated detection protocols in peer-reviewed literature (Supplementary Table S2). Reactions were performed using a CFX96 Touch Real-Time PCR System (Bio-Rad) and KAPA SYBR FAST One-Step enzyme mix (Roche). The reactions were incubated at 42°C for 10 min, 95°C for 3 min, followed by 40 cycles of 95°C for 10s, 60°C for 20s, and 72°C for 20s. Standard curves were prepared from purified viral amplification products and used to calculate absolute viral copy number in samples tested (Figures 1, 2).

## Results

### HTS diagnostic performance: a comprehensive sample processing and quality assessment

The field validation of Viroscope™ in collaboration with the Chilean phytosanitary authority (SAG) yielded comprehensive insights into viral pathogen prevalence within PEQ plant material, along with comparative metrics on the platform’s diagnostic performance.

From the initial specimens provided (*n* = 219), 144 (65.7%) met the stringent quality control parameters and were subjected to full bioinformatic analysis, providing a statistically robust dataset. After stringent quality control parameters, 65 specimens (29.6%) were discarded during preliminary assessment phases. Specifically, 32 samples exhibited low tissue integrity, (manifesting as pronounced oxidative degradation or necrotic zones upon morphological examination) while 33 specimens failed to meet RNA yield parameters (concentration higher than 70 ng/μL, RIN value <6.5) when analyzed via fluorometric quantification and capillary electrophoresis. The remaining 154 specimens underwent library construction protocols, incorporating validated controls including a characterized positive control specimen SS-S2 sweet cherry sample harboring multiple viral infections, notably *Prunus necrotic ringspot virus* (PNRSV) and an exogenous control matrix comprising *P. americana* tissue supplemented with synthetic viroid RNA for cross-contamination assessment. This approach enabled comprehensive assessment of potential cross-contamination events while establishing robust minimum threshold parameters for read assignment. Subsequently, a quality filtering criteria eliminated an additional 10 samples (5.6%). These samples failed to achieve the minimum sequencing depth threshold of 14 million paired-end reads, a parameter established through empirical validation to ensure adequate genome coverage for reliable viral detection. Thus the final analytical cohort consisted of 144 specimens, representing a statistically robust sample size for comprehensive viral surveillance across the targeted fruit tree species, as follows: *Prunus avium* (sweet cherry, *n* = 16), *Prunus domestica* (plum, *n* = 16), *Prunus salicina* (Japanese plum, *n* = 18), *Citrus* spp. (*n* = 3), *Fragaria × ananassa* (strawberry, *n* = 20), *Malus domestica* (apple, *n* = 24), *Olea europaea* (olive, *n* = 20), and *Persea americana* (avocado, *n* = 27). Notably, the implementation of this stringent multi-tiered quality control framework resulted in variable retention rates across species, with *Prunus* spp. exhibiting the highest quality metrics (75% retention), while *Citrus* spp. samples demonstrated increased susceptibility to quality-related exclusion (33% retention). This differential quality distribution would have necessitated statistical adjustment in subsequent comparative analyses to account for potential sampling bias effects on species-specific viral detection rates. Thus, the integration of both biological and technical controls provided a traceable validation chain throughout the analytical process, from nucleic acid extraction through computational identification, thereby ensuring high confidence in the diagnostic results.

### Host-species distribution: regulatory and emergent virus and viroids pathogen detection by Viroscope™

Infection patterns varied across fruit tree species, with distinct prevalence rates. The complete breakdown of virus-host associations is presented in Table 1 and Supplementary Table S3. From the total analyses (*n* = 144 samples) 41 specimens demonstrated presence of viral or viroid entities, with pronounced host-specific distribution patterns. Only citrus, Japanese plums, and strawberries showed a total absence of viruses in the analyzed samples.

**Table 1:** List of total viruses and viroids identified by Viroscope™ in field samples.

	***Sweet Cherry***	***Plum***	***Apple***	***Olive***	***Avocado***	***Citrus***	***Japanese plum***	***Strawberry***	**Control Sample**
N° of analyzed samples	16	16	24	20	27	3	18	20	
Samples positive for at least one virus or viroid	12 (75%)	8 (50%)	3 (12%)	8 (40%)	10 (37%)	0 (0%)	0 (0%)	0 (0%)	
Detected Regulated Viruses [with functional evidence]									
Prune dwarf virus	4 [4]	-	-	-	-	-	-	-	-
Detected Emergent Viruses [with functional evidence]									
Apple stem grooving virus	-	-	2 [0]	-	-	-	-	-	-
Apple stem pitting virus	-	-	1 [0]	-	-	-	-	-	-
Citrus concave gum-associated virus	-	-	1 [1]	-	-	-	-	-	-
Cherry green ring mottle virus	-	-	-	-	-	-	-	-	(+)
Cherry necrotic rusty mottle virus	-	-	-	-	-	-	-	-	(+)
Cherry virus A	12 [12]	-	-	-	-	-	-	-	(+)
Little cherry virus 1	-	-	-	-	-	-	-	-	(+)
Olive leaf yellowing-associated virus	-	-	-	7 [7]	-	-	-	-	-
Olive viral satellite RNA	-	-	-	2 [*]	-	-	-	-	-
Persea americana alphaendornavirus 1	-	-	-	-	10 [10]	-	-	-	-
Plum bark necrosis stem pitting-associated virus	-	8 [6]	-	-	-	-	-	-	-
Prunus necrotic ringspot virus	-	-	-	-	-	-	-	-	(+)
Ti ringspot-associated emaravirus	2 [1]	-	-	-	-	-	-	-	-
Detected Emergent Viroids									
Apple Hammerhead Viroid	-	-	2	-	-	-	-	-	-

Summary of viral and viroid detection using RNAseq and Viroscope analysis from leaf tissue samples. Numbers inside boxes represent the number of plant samples positive for each virus or viroid, and numbers in square brackets show samples that are also positive for detection of functional viral evidence. The control sample was a sweet cherry leaf sample and contains the viruses indicated in plus signs. * = Satellite viral RNA are devoid of conventional viral functional elements.

*P. avium* exhibiting the highest viral prevalence (75%, *n* = 12/16). The predominant pathogen in cherry specimens was *Cherry virus A* (CVA), with consistent detection of functional replication elements validating biological activity. *P. domestica* demonstrated significant viral presence (50%, *n* = 8/16), primarily associated with *Plum bark necrosis stem pitting-associated virus* (PBNSPaV), with functional evidence detected in 6 of 8 positive cases. The *O. europaea* and *P. americana* specimens exhibited moderate infection rates (40 and 37% respectively), characterized by predominant detection of *Olive leaf yellowing associated virus 1* (OLYaV) and *Persea americana alphaendornavirus 1* (PaEV1) respectively. *M. domestica* presented the most complex virological profile despite showing the lowest infection rate (12%, n = 3/24), harboring multiple viral entities including *Apple stem grooving virus* (ASGV), *Apple stem pitting virus* (ASPV), *Citrus concave gum-associated virus* (CCGaV), and *Apple hammerhead viroid* (AHVd).

As shown in Table 1, regulated viruses were detected in only 2.8% of samples, while emergent viruses were present in 28.5%, highlighting the advantage of HTS for comprehensive surveillance beyond regulated pathogens. Notably, no quarantine-listed viruses or viroids were detected across the analyzed specimens, validating current phytosanitary standard protocols.

Regarding the regulated viral pathogens examination, the *Prune dwarf virus* (PDV) was positively detected in 4 sweet cherry (*P. avium*) samples. Remarkably, Viroscope™ exhibited exceptionally high viral genome completeness values ranging from 0.93 to 0.98, indicating near-complete genome reconstruction. The presence of functional replication elements in all PDV-positive specimens provided definitive evidence of viral viability. These results demonstrated the diagnostic framework’s capacity for high certainty and biologically informed results. The PDV, a commonly infecting stone fruits virus, was first detected in Chile in the early 90s. Although it is already a circulating virus in the country, it is still crucial to prevent its spread as, especially in cases of coinfection with other stone fruit viruses, it can severely impact the development and growth of the fruit tree (Herrera, 2014).

Additionally, ASGV, ASPV, and CVA were also detected. These viruses are globally reported with variable infection severity across pomaceous and stone fruit hosts (Fiore et al., 2016; Rott et al., 2017; Nabi et al., 2018).

On the other hand, the Viroscope™ analysis revealed that 41 specimens (28.5%) harbored emergent viral or viroid entities, with pronounced host-specific distribution patterns. Notably, the PBNSPaV has been globally reported with variable infection severity across pomaceous and stone fruit hosts (Marais et al., 2014; Xu et al., 2019). Despite the fact that this virus has been reported in Chilean territory, it is still considered emerging by the local phytosanitary authority.

The AHVd, OLYaV, and PaEV1 are all viral agents previously identified in apples, cherries, olives, and avocados, respectively, but for which no clear pathological phenotypes have been demonstrated due to their presence in mixed infections with other viruses (Gao et al., 2017; Nabi et al., 2018; Fontana et al., 2019; Roberts et al., 2023).

The functional and positive detection of *Ti ringspot-associated emaravirus* (TiRSaV) and *Citrus concave gum-associated virus* (CCGaV) was also reported for *Prunus* spp. specimens and in *M. domestica* specimens, respectively (Table 1). The TiRSAV is an emerging virus of ornamental plants (Olmedo-Velarde et al., 2019) whereas CCGaV has already been detected in apple trees in Brazil and initially characterized as a potential cause of concave gum in citrus (Navarro et al., 2018; Minutolo et al., 2021). This result clearly confirms the capability of the Viroscope™ pipeline of novel pathogenic threats (emergent viruses).

Opposite, the satellite viral RNA devoid of conventional viral functional elements and requires other helper viruses to replicate and complete their viral cycle. All satellite viruses that infect plants are positive-sense single-stranded (+) RNA and classified into genera *Albetovirus*, *Aumaivirus*, *Papanivirus*, and *Virtovirus*. Since these infectious particles necessarily depend on helper determinants, their pathogenicity is conditioned to the viral co-infection of a host cell (Krupovic, 2021).

### Sensitivity performance assessment and method validation

**Figure 3:**
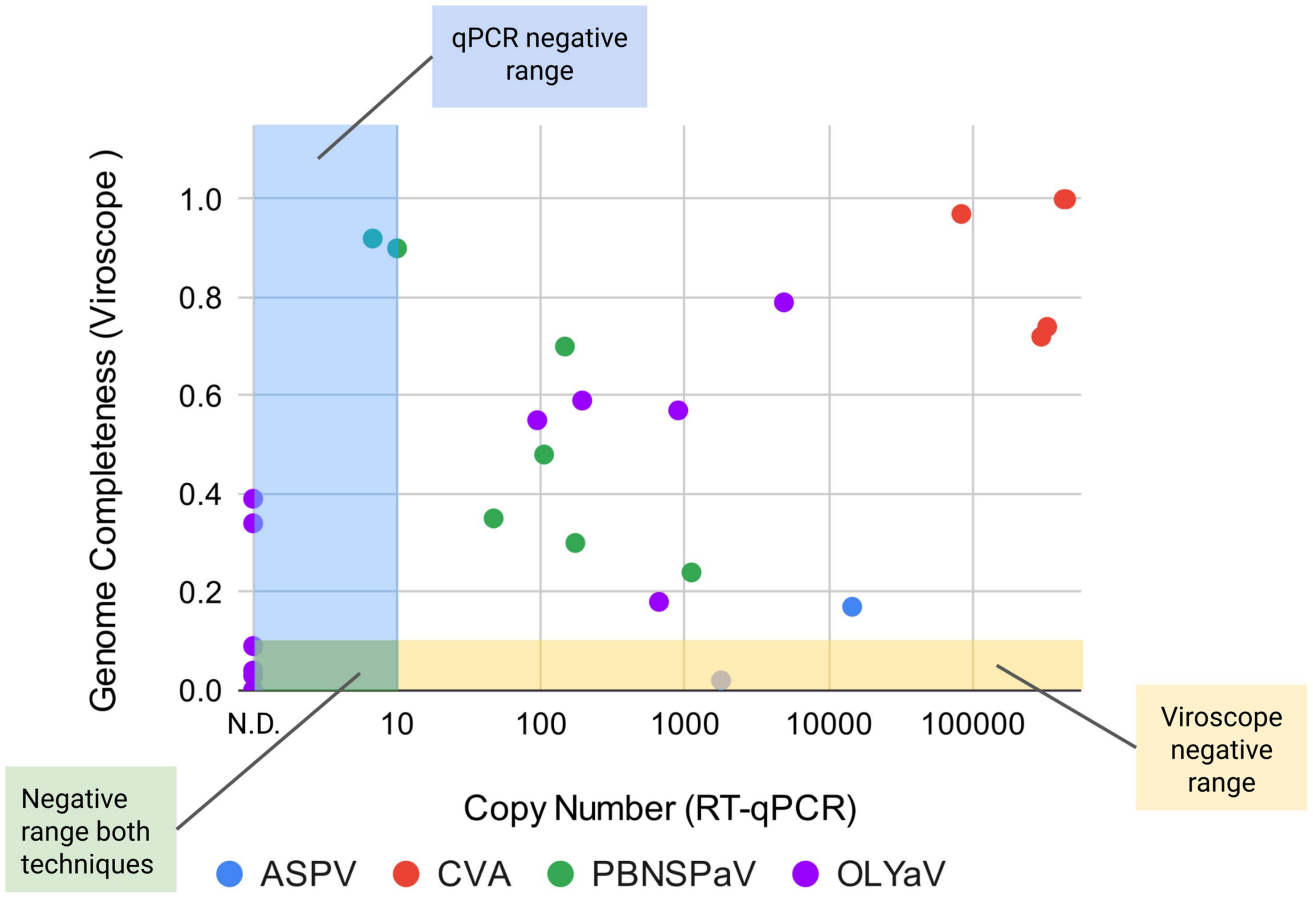
Comparison of viral detection using RNA seq and Viroscope™ versus RT-qPCR. Selected samples positive for viral detection using Viroscope™ algorithm were then subjected to RT-qPCR analysis. Viroscope™ calculated viral genome completeness was plotted against viral copy number detected by RT-qPCR. Colored regions indicate the negative detection ranges for each technology. Detailed information of samples can be found in Supplementary Table S4. N. D = not detected.

The comparative analysis between Viroscope™ and RT-qPCR (Figure 3 and Supplementary Table S4) was conducted targeting well-known viruses (ASPV, CVA, PBNSPaV, and OLYaV) and also representative of the phytosanitary post-quarantine entry scenario. The results revealed high diagnostic concordance (87.8%, 29/33 samples), with Viroscope™ demonstrating a superior detection capability in 9% of cases with viral signals below conventional RT-qPCR detection thresholds (3/33, for PBNSPaV and OLYaV). The correlation between viral genome assembly coverage and viral copy number (RT-qPCR) showed clear detection zones, with genome completeness values ranging from 0.1 to 1.0 plotted against RT-qPCR cycle threshold (Ct) values. The detection threshold analysis indicates optimal performance in the genome completeness range 0.1–0.9, correlating with moderate to high viral copy numbers in RT-qPCR positive samples. Thus, Viroscope™ exhibits enhanced sensitivity in marginal cases. Specifically, PBNSPaV detection revealed one sample exhibiting a viral signal below RT-qPCR detection threshold (10 copy number) yet positively identified by Viroscope™ with high genome completeness values and confirmed replicase presence. Similarly, OLYaV detection identified two specimens showing no RT-qPCR amplification but demonstrating clear viral presence via HTS (genome completeness values >0.4, functional evidence present). In this case, analysis of the OLYaV reads from these samples revealed they possess high sequence identity to the sequenced OLYaV V64 isolate (Ruiz-García et al., 2021). When the sequence of the RT-qPCR target ORF4 was analyzed in isolate V64, we found that several mutations (10 in total) are located in the primer annealing regions, suggesting that RT-qPCR detection of the V64 isolate with this primer set might have decreased sensitivity and only be feasible for higher viral loads, highlighting the advantages of non-directed methods of viral detection such as RNA seq. A single discordant result in which RT-qPCR detection was positive and Viroscope analysis was negative was seen for ASPV in a *M. domestica* sample. This was attributed to more stringent requirements for diagnosis as detection thresholds in Viroscope require a 10% of viral genome coverage, and in this case only a 2% was obtained (supplementary Table S4). The ASPV qPCR amplifies a 370 bp region, representing ~4% of the viral genome, which is still underneath Viroscope™ stringency. Overall, these results illustrate the importance of using unbiased and sensitive HTS-based approaches to avoid false negative results caused by the genetic variability of viruses in phytosanitary applications.

## Discussion

Field validation of Viroscope™, an HTS-based diagnostic tool for virus and viroid detection in quarantined plants, demonstrates its potential to transform current phytosanitary surveillance protocols through superior detection sensitivity, significantly reduced processing time, and reliable functional assessment of viral pathogens. This study extends the foundation established by Whattam et al. (2021) and Gauthier et al. (2022) by demonstrating the feasibility of HTS integration within a Latin American regulatory context, particularly significant as Chile is a major agricultural exporter with substantial economic interests in maintaining both international market access and robust phytosanitary protections (ODEPA, 2019; OECD, 2019; Central Bank of Chile, 2020). This transition to comprehensive molecular approaches establishes a valuable baseline for evidence-based phytosanitary decision-making and provides a unique regional-to-international perspective on agricultural sustainability, border security, and food supply chain integrity (FAO, 2024; IPPC, 2021; Melo et al., 2021).

The significance of these advances must be understood within the broader context of global economic impacts from plant viral pathogens. Notable examples include viruses like those associated with the cassava mosaic disease causing annual losses of $1.9–2.7B in African countries, and Citrus tristeza virus that has historically destroyed approximately 100 million trees globally (Moreno et al., 2008; Folimonova and Sun, 2022). Similarly, maize lethal necrosis disease demonstrated its severity in Kenya with losses of $52 M in a single season, and tomato yellow leaf curl disease can cause complete crop failure in susceptible cultivars (Jones, 2021). This situation is particularly critical considering the 65% increase in global plant trade volume from 2015–2020, which has intensified pressure on existing border control measures (WTO, 2022). While harmonization efforts have progressed—the successful alignment of HTS protocols might reduce quarantine processing times— significant barriers persist across major trading blocs (Alcalá Briseño et al., 2023; Whattam et al., 2021; Villamor et al., 2019; Rodoni, 2009). The quarantine facilities usually face mounting complexity in managing diverse plant species and pathogens, with testing costs ranging from $300–1,000 per sample depending on pathogen combinations (Adams et al., 2018; Maree et al., 2018; SAG, *personal communication*). In this challenging context, our validation of Viroscope™ sought to address fundamental performance criteria for effective phytosanitary tools. In the current PEQ scheme, plants typically undergo 6–24 months of quarantine with 3–5 sequential diagnostic tests. Our data demonstrate that comprehensive viral profiling through HTS can be completed within 10–15 days with superior diagnostic sensitivity, potentially reducing quarantine periods by 30–50%. This estimate is supported by preliminary implementation data from Australia and New Zealand (Maina and Jones, 2023; Whattam et al., 2021) and from internal collaborative trials with the Chilean phytosanitary agency showing that early detection permits expedited decision-making on quarantine release or extended monitoring. For high-value germplasm and commercial varieties, this acceleration represents significant economic benefits for both regulatory agencies and agricultural stakeholders.

In the tested plant material, the algorithm enabled unbiased detection of viral-pathogen genomes with high certainty assignment to corresponding viruses and viroids. From 144 samples corresponding to post-entry quarantine plants, no quarantine viruses or viroids were detected while 2.8% demonstrated regulated viruses and 28.5% contained emergent viruses. More interestingly, 25% of plants harbored emergent viruses with functional evidence. These findings represent a significant step toward the implementation of HTS methodologies during PEQ schemes in Chile, aligning with international border biosecurity and surveillance applications being developed in leading countries (Bag et al., 2015; Rott et al., 2017; Whattam et al., 2021; Gauthier et al., 2022; Lelwala et al., 2022; Delmiglio et al., 2023). To ensure reliable implementation, we established rigorous technical standards aligned with international best practices. The economic advantages of Viroscope™ implementation are substantial when considering the complete diagnostic process. Traditional diagnostic approaches require multiple sequential tests for comprehensive viral screening. In Chile, while individual RT-qPCR assays are cost-effective at approximately $17–20 per reaction, the cumulative expense for comprehensive pathogen screening in quarantine contexts (including multiple target pathogens, extraction procedures, labor and confirmatory testing), typically ranges from $200–400 per plant sample processed through the full diagnostic workflow (i.e., considering an average of 10 molecular targets of interest per plant sample at PEQ). In contrast, the HTS-based Viroscope™ approach provides a single-test solution with comparable total costs while significantly increasing diagnostic scope and certainty. For regulatory agencies, this might represent a potential cost reduction while increasing diagnostic scope and certainty.

Our study confirms that standardized operating procedures are essential for implementing HTS as part of routine pathogen screening practices (Maree et al., 2018; Lebas et al., 2022; Massart et al., 2022; Rong et al., 2023; Bester and Maree, 2024). The integration of rigorous bioinformatics analysis algorithms and establishment of clear detection thresholds were key to achieving sensitive and accurate identification of viruses present in samples (Bag et al., 2015; Piper et al., 2019; Ning et al., 2024). The analytical sensitivity and specificity demonstrated by Viroscope™ significantly surpasses traditional molecular approaches. While conventional RT-PCR methods detect specific viral targets with high sensitivity, they are inherently limited by primer design constraints and the requirement for prior sequence knowledge (Boonham et al., 2014; Mehetre et al., 2021). In contrast, the untargeted HTS-based approach enables simultaneous detection of multiple pathogens, including divergent variants and novel species, addressing a critical need in quarantine testing (Maree et al., 2018; Massart et al., 2022). It is important to note that while our study focuses on detection rather than pathogenicity confirmation, the biological significance of our findings is supported by the identification of viral replicase transcripts, which provides evidence of functionality. This biologically-informed approach distinguishes between potentially infectious viral entities and non-viable genomic fragments, a critical distinction for regulatory decision-making. For confirmed detections, standardized phytosanitary protocols would typically follow, including pathogenicity assessment through biological indexing and epidemiological surveillance in accordance with IPPC standards.

The direct comparative analysis between Viroscope™ and RT-qPCR methods (the current gold standard) showed that our RNA-seq based approach had a high degree of concordance with RT-qPCR results (87.8% of compared samples). Furthermore, HTS exhibited more sensitivity than qPCR for two different viruses, detecting viral signals in samples with genome completeness values >0.3 that were below conventional RT-qPCR detection thresholds (particularly, for PBNSPaV and OLYaV). This enhanced sensitivity addresses a critical vulnerability in traditional diagnostic protocols, where low-titer viral infections might escape detection during quarantine testing. The unique discrepancy between both methods was observed in the case of Cherry leaf roll virus (CLRV, unpublished data). A follow-up analysis performed by our laboratory revealed three contributing factors: (1) tissue-specific viral distribution differences–SAG protocols examined bud tissue while our study analyzed leaves from the same plants, with quantitative viral distribution assays confirming 5–10 fold higher viral titers in bud tissue for this particular virus; (2) primer design variations—sequence analysis identified two distinct CLRV variants in Chilean olive populations with 8–12% sequence divergence in common primer binding regions; and (3) potential RT-qPCR false positives, particularly in high-cycle threshold cases (Ct > 35) which statistical analysis identified as falling in the ambiguous detection zone. These findings underscore the importance of standardized sampling protocols and highlight how complementary diagnostic approaches can provide more comprehensive viral surveillance. The implementation of alien controls for cross-contamination monitoring provided an adaptive detection threshold framework that significantly reduces false positives while maintaining high analytical sensitivity. The operation of HTS protocols in a high-throughput format enabled processing of 70 samples bi-weekly without compromising quality metrics (Bester et al., 2021; Massart et al., 2022). While automation represents an optional enhancement to the workflow, the core Viroscope™ methodology maintains its high performance and reliability even with manual processing. When implemented, automation capabilities might reduce labor costs by an estimated 45% and decrease processing time from sample collection to final diagnostic report.

Indeed, previous concerns regarding HTS result interpretation and robust bioinformatic frameworks have been significant barriers to regulatory adoption (Massart et al., 2017; Massart et al., 2022). The Viroscope™ pipeline addresses these challenges through several key innovations. First, careful optimization of bioinformatic parameters, including coverage thresholds, cross-contamination monitoring, and functional annotation criteria, provides reliable and reproducible results suitable for regulatory decision-making (Maree et al., 2018; Rott et al., 2017). Second, the integration of functional annotation analysis -using metrics based on viral replicase transcripts correlated with genome completeness values- adds biological context to detection events, enhancing diagnostic certainty in cases of low viral titers (Valenzuela et al., 2022). Third, the cloud-based automated data analysis workflow incorporates consensus-based validation standards and quality control metrics that ensure reproducibility across different sequencing runs and laboratory settings. This comprehensive computational framework transforms raw sequencing data into actionable diagnostic information, without requiring extensive bioinformatics expertise (Valenzuela et al., 2022). These technical innovations translate directly into practical regulatory applications with substantial implications. A recent comprehensive review on HTS implementation in plant health certification has highlighted the need for standardized approaches to data analysis and interpretation (Maina et al., 2024). The authors emphasize that despite technical advances, the lack of harmonized bioinformatic frameworks and clear detection thresholds remains a significant barrier to widespread adoption. To note, several bioinformatic tools currently support HTS-based viral diagnostics, including PVDP (Gutiérrez et al., 2021), VirFinder (Ho and Tzanetakis, 2014), GA-VirReport (Lelwala et al., 2022), and PhytoPipe (Hu et al., 2023). Viroscope™ distinguishes itself through its integration of functional annotation via viral replicase identification, which significantly enhances diagnostic confidence beyond coverage metrics alone. Unlike systems requiring local bioinformatic expertise, its cloud-based implementation provides immediate accessibility while maintaining compatibility with both short and long-read sequencing. Comparative validation studies demonstrate superior diagnostic accuracy (>99.9% sensitivity/specificity) compared to typical ranges (90–95%) reported for alternative platforms. Most importantly, our field validation study showed equivalent or superior viral detection capacity to current established methods, which might enable expedited decision-making on quarantine release or extended monitoring.

From a practical implementation perspective, our study directly addresses two critical stages of the approach to comply with phytosanitary requirements: enhancing collaboration among regulatory agencies in standard enforcement and promoting investment in innovative diagnostic technologies. Viroscope™ provides high-certainty diagnoses through comprehensive genome analysis and functional annotation, overcoming traditional PCR limitations such as primer design constraints and cross-reactivity issues. While RT-qPCR remains valuable for targeted detection of regulated viral pathogens, HTS implementation offers a powerful complementary tool for comprehensive phytosanitary surveillance, particularly for emergent and variant viral strains. Notably, it reduces false negative rates through enhanced detection of low-titer viral infections and/or by cases where viral genetic variability leads to failure of PCR amplification when primer annealing regions are mutated. This was demonstrated by successful identification in samples below RT-qPCR thresholds (9% of cases, n = 3/33). In addition, traditional PCR limitations such as primer design constraints and cross-reactivity issues (as evidenced in the ASPV/ASGV case where sequence homology >80% led to potential misidentification using targeted amplification). Thus, the complementary implementation of HTS is particularly valuable for emergent and variant viral strains that might escape traditional detection methods, offering a powerful tool for comprehensive phytosanitary surveillance (Olmos et al., 2018; Kutnjak et al., 2021).

It should be noted that the current Viroscope™ algorithm is an universal approach for viral detection, not only for RNA viruses but also for DNA viruses. For DNA viruses such as begomoviruses, identification by Viroscope™ is a direct evidence of functional activity as DNA viruses require transcription intermediates to produce viral components necessary for replication. The absence of an encoded replicase does not circumvent detection and functional activity, in this type of cases. High coverage values indicate potential active infections that should be considered in phytosanitary risk assessments. For comprehensive applications involving diverse viral taxa, complementary confirmation using traditional methods can be valuable. Additionally, the Viroscope™ framework will be extended to include geospatial integration for epidemiological monitoring across agricultural regions and other pathogen classes (primarily bacteria). While maintaining its core functional diagnostic approach, this modular expansion is currently under development, with the aim of providing phytosanitary authorities with coordinated early detection systems that enhance preventive surveillance capabilities.

Finally, our findings establish Viroscope™ as a powerful decision-making tool for plant virus border surveillance, enabling not only screening for regulated viral pathogens but also identification of potential emerging agents of plant diseases. This dual capability simultaneously strengthens phytosanitary protection while facilitating market competitiveness and global plant trade through more efficient and accurate diagnosis. While Viroscope™ is a proprietary platform, it is a published algorithm, specifically designed for integration into regulatory workflows through secure cloud-based implementation requiring no local installation, with standardized data formats compatible with existing phytosanitary information systems, and a framework for authorized diagnostic laboratories approved by regulatory authorities. This approach balances intellectual property with the practical requirements of phytosanitary agencies for accessible, reliable diagnostic tools.

## Conclusion

Border biosecurity and food supply are of global concern. HTS techniques ensure highly certain diagnoses and competitiveness in the agricultural market. International authorities urgently need to regulate HTS protocols for phytosanitary purposes. This work establishes a comprehensive framework for implementing HTS technologies in regulatory scenarios. Viroscope™, a validated and standardized HTS-based pipeline for virus and viroid detection of high certainty, is instrumental in our efforts to ensure food safety from plant viral pathogens. Viroscope.io facilitates adoption of these techniques by making HTS interpretation accessible for phytosanitary agencies worldwide. Future studies should focus on expanding the molecular validation of HTS to additional plant pathogen species (i.e., Bacteria), which would further strengthen its application in global plant health management. The successful collaboration between research institutions and regulatory agencies demonstrated in this study provides a model for effectively bridging scientific innovation with practical regulatory implementation. As international plant trade continues to increase in volume and complexity, integrated approaches combining advanced molecular diagnostics with streamlined regulatory frameworks will be essential for balancing agricultural market competitiveness with robust phytosanitary protection.

## Data Availability

The original contributions presented in the study are publicly available. This data can be found at: https://www.ncbi.nlm.nih.gov/bioproject/PRJNA1249445.

## Footnotes

^1^ support.illumina.com/

## Supplementary material

Supplementary Figure S1 Genome completeness and assigned reads for the SS-S2 sample at different sequencing depths. Reads from the SS-S2 sample were randomly subsampled and analyzed using the Viroscope™ algorithm. Plot A displays genome completeness, while plot B shows the number of assigned reads at different sequencing depths. The green line represents the coverage threshold of 10% completeness. CGRMV, *Cherry Green Ring Mottle Virus*; CNRMV, *Cherry Necrotic Rusty Mottle Virus*; CVA, *Cherry Virus A*; LChV-1, *Little Cherry Virus* 1; PNRSV, *Prunus Necrotic Ringspot Virus*.

Supplementary Figure S2 Genome completeness and assigned reads for the VdS_S1 sample at different sequencing depths. Reads from the VdS_S1 sample were randomly subsampled and analyzed using the Viroscope™ algorithm. Plot A displays genome completeness, while plot B shows the number of assigned reads at different sequencing depths. The green line represents the coverage threshold of 40% completeness. The VdS_S1 sample corresponds to a positive control for *Citrus dwarfing viroid*, *Citrus exocortis viroid*, and *Hop stunt viroid*.

Supplementary Table S1 Regulated plant viral pest list under the local phytosanitary authority (SAG). The list only refers to the species of major fruit crops subjected to post-entry quarantine monitoring in Chile, during the period of this study (January-April 2023).

Supplementary Table S2 Primer list used in qPCR methods for sensitivity analysis validation.

Supplementary Table S3 Host-specific distribution of virus and viroid detections across all sequenced specimens. This dataset shows a comprehensive analysis of 144 post-entry quarantine plant samples across eight economically significant fruit crops. For each sample, host species identification and sample ID are provided. Viral detections are denoted indicating de VGAC value, with specimens categorized by threshold metrics (≥10% genome coverage for viruses; ≥40% for viroids) as previously described in the Materials and Methods section. Functional evidence assessment through viral replicase identification is indicated in boldface VGAC value, when applicable. Due to the large size of the table, we are providing the official upload link from the submission system, through which the file has been made available. https://www.frontiersin.org/api/v4/articles/1609663/file/Table_3.xlsx/1609663_table_3/1.

Supplementary Table S4 List of samples used to compare viral detection between RNA seq and ViroscopeTM versus RT-qPCR. Plant species, sample code, and values of RNA seq Viroscope and qRT-PCR detection are shown, plus details of other detected virus or viroids in the sample.
