# Supplementary material for: Viroscope™: a universal solution for plant virus and viroid diagnostics using HTS and cloud-based analysis
Source: Front Microbiol. 2025 Jul 3;16:1609663. doi: 10.3389/fmicb.2025.1609663 (PMC12267180; doi:10.3389/fmicb.2025.1609663)
Supplement: Supplementary Figure S1 — Genome completeness and assigned reads for the SS-S2 sample at different sequencing depths. Reads from the SS-S2 sample were randomly subsampled and analyzed using the Viroscope™ algorithm. Plot A displays genome completeness, while plot B shows the number of assigned reads at different sequencing depths. The green line represents the coverage threshold of 10% completeness. CGRMV, Cherry Green Ring Mottle Virus; CNRMV, Cherry Necrotic Rusty Mottle Virus; CVA, Cherry Virus A; LChV-1, Little Cherry Virus 1; PNRSV, Prunus Necrotic Ringspot Virus. [file Table_1.docx]

**Supplementary Tables**

**Table S1. Regulated plant viral pest list under the local phytosanitary authority (SAG)**

| Quarantine Viruses and Viroids |
| --- |
| *Avocado sunblotch viroid* |
| *Blueberry leaf mottle virus* |
| *Blueberry scorch virus* |
| *Blueberry shock virus* |
| *Cherry leaf roll virus* |
| *Cherry rasp leaf virus* |
| *Citrus tatter leaf virus* |
| *Grapevine pinot gris virus* |
| *Grapevine red blotch virus (Sin.: Grapevine red blotch-associated virus)* |
| *Grapevine vein clearing virus* |
| *Little cherry virus 2* |
| *Peach mosaic virus* |
| *Peach rosette mosaic virus* |
| *Plum pox virus (except PPV-D)* |
| *Raspberry leaf curl virus* |
| *Raspberry ringspot virus* |
| *Tomato black ring virus* |
| *Tomato bushy stunt virus* |
| *Tomato leaf curl New Delhi virus* |
| Regulated Viruses |
| *Apple chlorotic leaf spot virus* |
| *Apple mosaic virus* |
| *Arabis mosaic virus* |
| *Plum pox virus raza D* |
| *Prune dwarf virus* |
| *Prunus necrotic ringspot virus* |
| *Strawberry crinkle virus* |
| *Strawberry latent ringspot virus* |
| *Strawberry mild yellow edge virus* |
| *Strawberry mottle virus* |
| *Tomato ringspot virus* |

**Table S2. Primer list used in qPCR methods for sensitivity analysis validation**

| Virus | Fwd Primer | Rev Primer | Reference |
| --- | --- | --- | --- |
| ASPV | ATGTCTGGAACCTCATGCTGCAA | TTGGGATCAACTTTACTAAAAGCATAA | Nabi et al., 2018 |
| CVA | ACATCTGACTGCGAAACCAG | TGTTCGCTTGTACCCATCAC | Valenzuela et al., 2022 |
| PBNSPaV | TTCCGTCGGCCGTTATACC | CCAGCCACAAACTTGAAGG | Valenzuela et al., 2022 |
| OLYaV | GGGACGGTTACGGTCGAGAG | CGAAGAGAGCGGCTGAAGGCTC | Sabanadzovic et al., 1999 |
