# Supplementary material for: Viroscope™: a universal solution for plant virus and viroid diagnostics using HTS and cloud-based analysis
Source: Front Microbiol. 2025 Jul 3;16:1609663. doi: 10.3389/fmicb.2025.1609663 (PMC12267180; doi:10.3389/fmicb.2025.1609663)
Supplement: Supplementary Table S3 — Host-specific distribution of virus and viroid detections across all sequenced specimens. This dataset shows a comprehensive analysis of 144 post-entry quarantine plant samples across eight economically significant fruit crops. For each sample, host species identification and sample ID are provided. Viral detections are denoted indicating de VGAC value, with specimens categorized by threshold metrics (≥10% genome coverage for viruses; ≥40% for viroids) as previously described in the Materials and Methods section. Functional evidence assessment through viral replicase identification is indicated in boldface VGAC value, when applicable. Due to the large size of the table, we are providing the official upload link from the submission system, through which the file has been made available. https://www.frontiersin.org/api/v4/articles/1609663/file/Table_3.xlsx/1609663_table_3/1. [file Table_3.docx]

| **Plant species** | **Sample code** | **Target** | **Viroscope Completeness** | **Viroscope detection** | **RT-qPCR copy number** | **RT-qPCR detection** | **Other virus and viroids in sample** |
| --- | --- | --- | --- | --- | --- | --- | --- |
| **Apple** | 11335 | ASPV | 0.02 | – | 1,779 | + | - |
|  | 11337 | ASPV | 0.17 | + | 14,493 | + | ASGV, AHVd |
| **Plum** | 11311 | PBNSPaV | 0 | – | N.D. | – | - |
|  | 11312 | PBNSPaV | 0.92 | + | 6.8 | – | - |
|  | 11313 | PBNSPaV | 0.7 | + | 146 | + | - |
|  | 11314 | PBNSPaV | 0.24 | + | 1,109 | + | - |
|  | 11315 | PBNSPaV | 0.3 | + | 173 | + | - |
|  | 11316 | PBNSPaV | 0.48 | + | 105 | + | - |
|  | 11317 | PBNSPaV | 0.9 | + | 10 | + | - |
|  | 11318 | PBNSPaV | 0.35 | + | 46 | + | - |
| **Sweet Cherry** | 29352 | CVA | 0 | – | N.D. | – | - |
|  | 29353 | CVA | 0 | – | N.D. | – | - |
|  | 29354 | CVA | 0 | – | N.D. | – | - |
|  | 29355 | CVA | 0 | – | N.D. | – | - |
|  | 29356 | CVA | 1 | + | 427,308 | + | - |
|  | 29357 | CVA | 1 | + | 443,769 | + | - |
|  | 29358 | CVA | 0.74 | + | 327,965 | + | PDV |
|  | 29359 | CVA | 0.72 | + | 298,392 | + | PDV |
|  | 29361 | CVA | 0.97 | + | 83,056 | + | - |
| **Olive** | 21174-1 | OLYaV | 0.59 | + | 193 | + | - |
|  | 21174-2 | OLYaV | 0.55 | + | 94 | + | - |
|  | 21174-3 | OLYaV | 0.09 | – | N.D. | – | - |
|  | 21178-1 | OLYaV | 0.39 | + | N.D. | – | OL-sat-RNA |
|  | 21178-2 | OLYaV | 0.34 | + | N.D. | – | - |
|  | 21179-1 | OLYaV | 0 | – | N.D. | – | - |
|  | 21179-2 | OLYaV | 0 | – | N.D. | – | - |
|  | 21181-1 | OLYaV | 0.18 | + | 659 | + | - |
|  | 21181-2 | OLYaV | 0 | – | N.D. | – | - |
|  | 21183-1 | OLYaV | 0.04 | – | N.D. | – | - |
|  | 21183-2 | OLYaV | 0 | – | N.D. | – | - |
|  | 21183-3 | OLYaV | 0.03 | – | N.D. | – | OL-sat-RNA |
|  | 72784-1 | OLYaV | 0.79 | + | 4,869 | + | - |
|  | 72784-2 | OLYaV | 0.57 | + | 897 | + | - |

N.D = Not detected
